# Tropical cyclone rainfall area controlled by relative sea surface temperature

**DOI:** 10.1038/ncomms7591

**Published:** 2015-03-12

**Authors:** Yanluan Lin, Ming Zhao, Minghua Zhang

**Affiliations:** 1 Ministry of Education Key Laboratory for Earth System Modeling, Center for Earth System Science, Tsinghua University, Beijing 100084, China; 2 University Corporation for Atmospheric Research/Geophysical Fluid Dynamics Laboratory, Princeton, New Jersey 08540-6649, USA; 3 School of Marine and Atmospheric Sciences, Stony Brook University, Stony Brook, New York 11794-5000, USA

## Abstract

Tropical cyclone rainfall rates have been projected to increase in a warmer climate. The area coverage of tropical cyclones influences their impact on human lives, yet little is known about how tropical cyclone rainfall area will change in the future. Here, using satellite data and global atmospheric model simulations, we show that tropical cyclone rainfall area is controlled primarily by its environmental sea surface temperature (SST) relative to the tropical mean SST (that is, the relative SST), while rainfall rate increases with increasing absolute SST. Our result is consistent with previous numerical simulations that indicated tight relationships between tropical cyclone size and mid-tropospheric relative humidity. Global statistics of tropical cyclone rainfall area are not expected to change markedly under a warmer climate provided that SST change is relatively uniform, implying that increases in total rainfall will be confined to similar size domains with higher rainfall rates.

## Abstract (web summary)

The rainfall rate of tropical cyclones is expected to increase under a warmer climate, yet likely changes in rainfall area remain unknown. Here, the authors combine satellite data and model simulations and show that rainfall area is dependent on relative sea surface temperatures.

## Graphical abstract

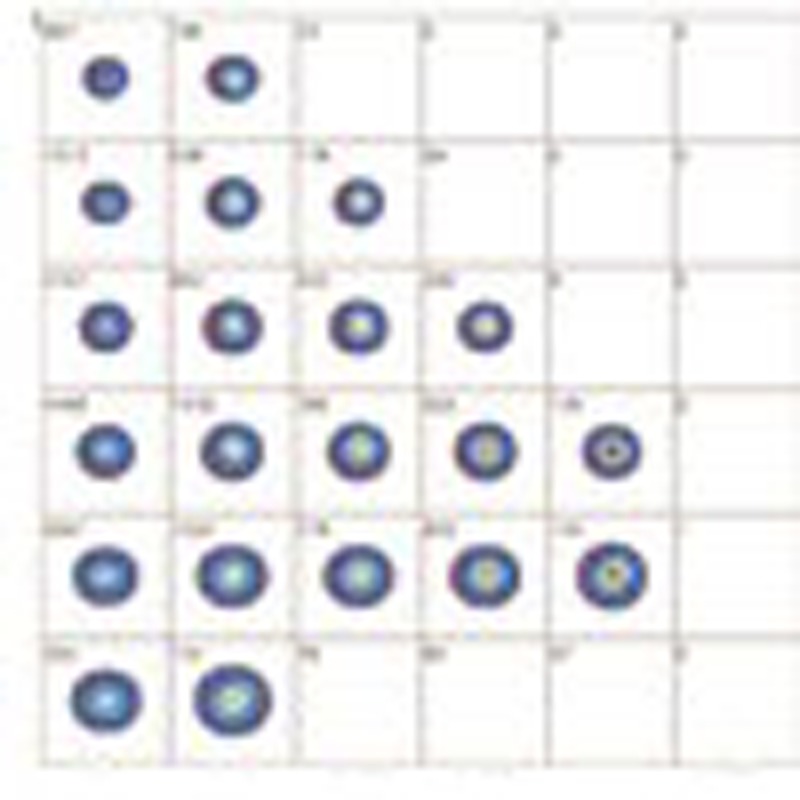

Tropical cyclones (TCs) induce heavy precipitation and flooding in addition to damages associated with high winds and storm surges^1^. Precipitation associated with TCs accounts for ~6–9% of total precipitation over the Tropics on average, but can account for as much as 50% of total precipitation over large portions of ocean basins^2^. The cumulative rainfall and potential damages associated with a TC depend in large part on the rainfall rate, rainfall area coverage and translation speed of the storm. Past studies have investigated potential changes in the frequency and rainfall intensity of TCs under a warmer climate. These studies project a decrease in the total number of TCs but an increase in the occurrence frequency of stronger TCs^3^,^4^,^5^,^6^. One of these studies also projects an ~20% increase in TC precipitation within 100 km of the storm centre by the late 21st century^6^. Few studies have examined possible changes in TC rainfall area. TC rainfall area is closely related to the TC wind field, with heavy precipitation generally confined within the outermost closed isobar^7^,^8^. TC rainfall area is therefore a gross measure of TC size, particularly with respect to the outer radius^8^. Note that this definition of TC size can differ from definitions based on the TC wind field, such as the radius of maximum wind or gale force wind. Rainfall area or size can be influenced by a number of factors, such as environmental humidity, low-level vorticity, vertical wind shear, TC latitude and TC intensity^9^,^10^,^11^,^12^,^13^,^14^,^15^,^16^,^17^,^18^,^19^. It varies greatly from one TC to another. Theoretical studies and idealized radiative convective equilibrium simulations have suggested that TC potential intensity (PI) divided by the Coriolis parameter may be a good measure of TC size^14^,^15^,^16^,^17^; however, the extent to which this scaling is realized in the real world remains uncertain^18^,^19^. Key questions remain regarding the mechanisms that control TC size and its changes in a warming climate.

Using currently available satellite measurements and global atmospheric model simulations, we examine the sea surface temperature (SST) dependence of rainfall area and rainfall rate in TCs. We find that TC rainfall area is controlled primarily by the relative magnitude of SST in the TC local environment with respect to the tropical mean SST (that is, the relative SST), while TC rainfall rate increases with absolute SST. TC rainfall area is therefore not likely to change markedly in a warmer climate provided that SST changes are relatively uniform throughout the tropics. Increases in TC rainfall are then mostly attributable to increases in TC rainfall rate. The strong dependence of TC rainfall area on relative SST is consistent with previous numerical simulations that suggested a tight relationship between TC size and mid-tropospheric relative humidity (RH) in the TC environment. In the tropics, relative SST strongly regulates the spatial distribution of mid-tropospheric humidity. TCs tend to expand as they move into regions where the mid-tropospheric humidity is high, which closely correspond to regions of high relative SST. Although the impact of relative SST can be partly understood using the PI framework, our analyses of global atmospheric model simulations suggest that relative SST is a better predictor of TC rainfall area than PI.

## Results

### TC rainfall characteristics

We determine TC rainfall area using two objective methods (see Methods) based on 11 years of satellite precipitation retrievals (the Tropical Rainfall Measuring Mission, or TRMM, 3B42 data set)^20^ and 21 years of cloud brightness temperatures (Cloud Archive User Services, or CLAUS)^21^ in tandem with observed TC tracks from the International Best Track Archive for Climate Stewardship (IBTrACS)^22^. We use these data to study the climatic controls on TC rainfall area. We use TC precipitation statistics from Geophysical Fluid Dynamics Laboratory (GFDL) high-resolution atmospheric model (HIRAM)^23^ simulations at a 50-km resolution both to corroborate our observational findings and to examine the potential impacts of global warming induced by increasing concentrations of anthropogenic greenhouse gases on TC rainfall and size.

**Figure 1:**
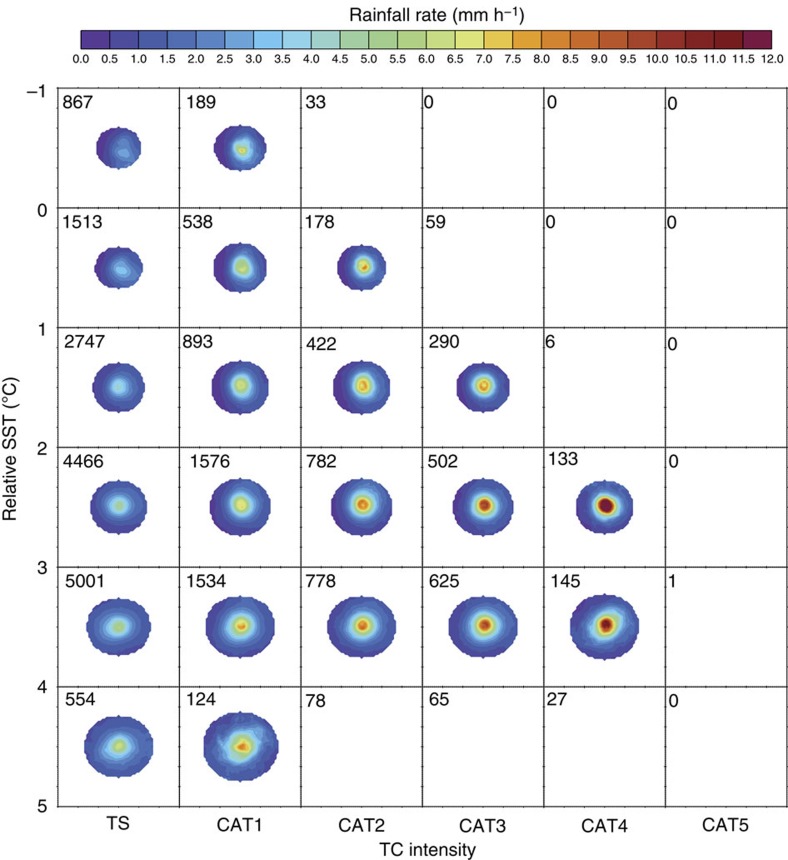
Average rainfall distribution composited by relative SST and intensity. Rainfall rate (colour shaded in mm h^−1^) composited by relative SST (*y*-axis) and tropical cyclone intensity (*x*-axis, from tropical storm (TS) to category 5 (CAT5)). The number in the upper left in each box denotes the number of samples in each composite. Only composites with sample sizes >100 are shown. For each composite, pixels with occurrence frequencies <50% are omitted.

**Figure 2:**
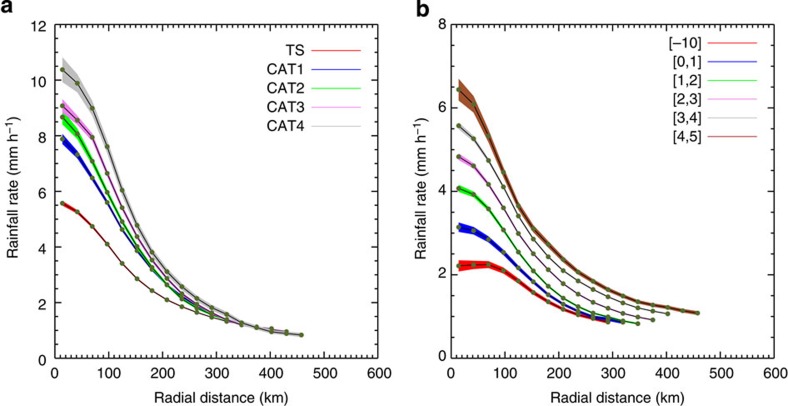
Radial distribution of azimuthally averaged rainfall rate. (**a**) Radial profiles of rainfall rate for tropical cyclones of different intensities (from tropical storm (TS) to category 4 (CAT4)) in the [3 °C, 4 °C] relative SST bin. (**b**) Radial profiles of rainfall rate for tropical storms within different relative SST bins. Shading indicates one s.e.m.

TC rainfall distributions and areas (see Methods) are stratified according to different categories of TC intensity and relative SST, where relative SST is defined as the SST in the TC environment minus the tropical (30°S-30°N) mean SST (Fig. 1). The relationship between TC intensity and TC size (the horizontal direction in Fig. 1) is weak, consistent with previous results^9^. By contrast, there is a robust increase in the average TC rainfall area with increasing relative SST in all intensity categories (the vertical direction in Fig. 1). The dependence of rainfall rate on intensity and relative SST is further illustrated in Fig. 2, which shows the radial distributions of azimuthally averaged rainfall rate for storms of different intensities within the [3 °C,4 °C] relative SST bin (row 5 in Fig. 1) and for tropical storms in different relative SST bins (column 1 in Fig. 1). As expected, TC rainfall rate increases markedly with TC intensity, especially towards the storm centre, but rainfall radius (defined as the radius where the azimuthally averaged rainfall rate reaches a threshold rainfall rate, see Methods) changes little with TC intensity (Fig. 2a). By contrast, high rainfall rates expand outwards to larger radii as relative SST increases (Fig. 2b). Increases in rainfall area with relative SST mean that total TC rainfall also increases with relative SST.

### Dependence of TC rainfall area and rate on SST

**Figure 3:**
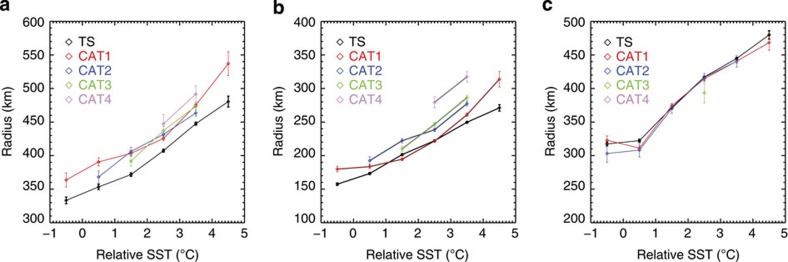
Variation of tropical cyclone rainfall radius with relative SST. (**a**) Average tropical cyclone rainfall radii in the specified one-degree relative SST bins based on TRMM data using the first method (see Methods for details). Different colours indicate sets of tropical cyclones of different intensities, from tropical storm (TS) to category 4 (CAT4). (**b**) Same as (**a**) but based on CLAUS data using the second method. (**c**) Same as (**a**) but based on the HIRAM AMIP simulation using the first method. Error bars indicate one s.e.m.

TC rainfall area determined using both objective methods is typically largest in the Western North Pacific and smallest in the Eastern Pacific. This result is consistent with geographic variations in TC size metrics based on wind fields^9^,^18^,^19^. The mean TC rainfall radius increases by ~100 km as relative SST increases from the [0 °C,1 °C] bin to the [3 °C,4 °C] bin (Fig. 3a). This result is qualitatively robust for all categories of TCs. Analysis of rainfall radii determined using an alternative objective method based on brightness temperatures also shows an increase in rainfall radius with relative SST (Fig. 3b), although the mean radii are smaller because of differences in the methodology (See Methods). Furthermore, this dependence of TC rainfall size on relative SST is reproduced in an Atmospheric Model Intercomparison Project (AMIP)-type (that is, using historical SSTs and sea ice as boundary conditions) simulation using HIRAM (Fig. 3c). The success of the model in simulating the TC size dependence suggests that the underlying mechanism may be relatively simple and controlled by large-scale atmospheric conditions rather than cumulus-scale dynamics. The strong dependence of TC size on relative SST can also be identified in an analysis of absolute SST, although the strength of the regression is slightly degraded (Supplementary Fig. 1). Temporal fluctuations in tropical mean SST are relatively small, so that changes in relative and absolute SST are largely coherent. Both relative and absolute SST reflect regional SST variations in the present climate. We are therefore unable to differentiate between relative and absolute SST impacts on TC rainfall area based on observations alone.

Two pairs of HIRAM experiments were conducted to examine the dependence of TC rainfall area and rate on relative and absolute SST. The first pair contrasts a 25-year control simulation with prescribed climatological (seasonally-varying) SSTs and sea ice with a perturbed simulation in which a uniform 2 K warming (relative to the control simulation) is imposed (P2K). The second pair contrasts an AMIP-type simulation that uses historical SSTs and sea ice from 1981 to 2009 as the lower boundary conditions with a simulation in which a uniform 4 K warming (relative to the AMIP simulation) is imposed (P4K). Global warming simulations use the same CO_2_ concentration as the control to isolate the SST impacts from CO_2_ impacts^24^. Absolute SSTs increase by 2 and 4 K in the P2K and P4K simulations, but relative SSTs stay the same as the control and AMIP simulations. TC genesis locations and tracks are broadly similar in all of these simulations (Supplementary Fig. 2).

**Figure 4:**
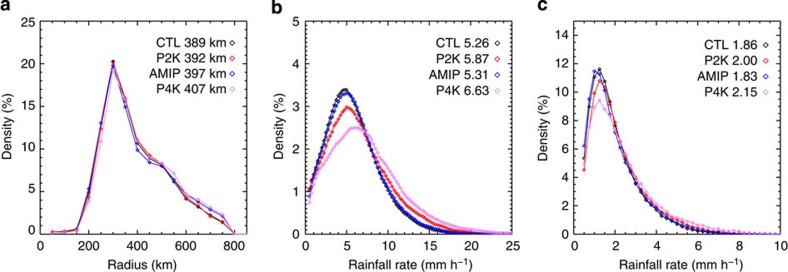
Frequency distributions of rainfall radius and rainfall rate from HIRAM simulations. (**a**) Frequency distributions of TC rainfall radius from the four HIRAM simulations (CTL=the control simulation; AMIP=the AMIP simulation; P2K=the P2K simulation and P4K=the P4K simulation; see text for details). (**b**) Frequency distributions of TC rainfall rate within a 100-km radius of the TC centre. (**c**) Same as (**b**) but for TC rainfall rate within the objectively determined rainfall radius. Mean values of rainfall radius (in km) and rainfall rate (in mm h^−1^) are labelled in each panel.

**Figure 5:**
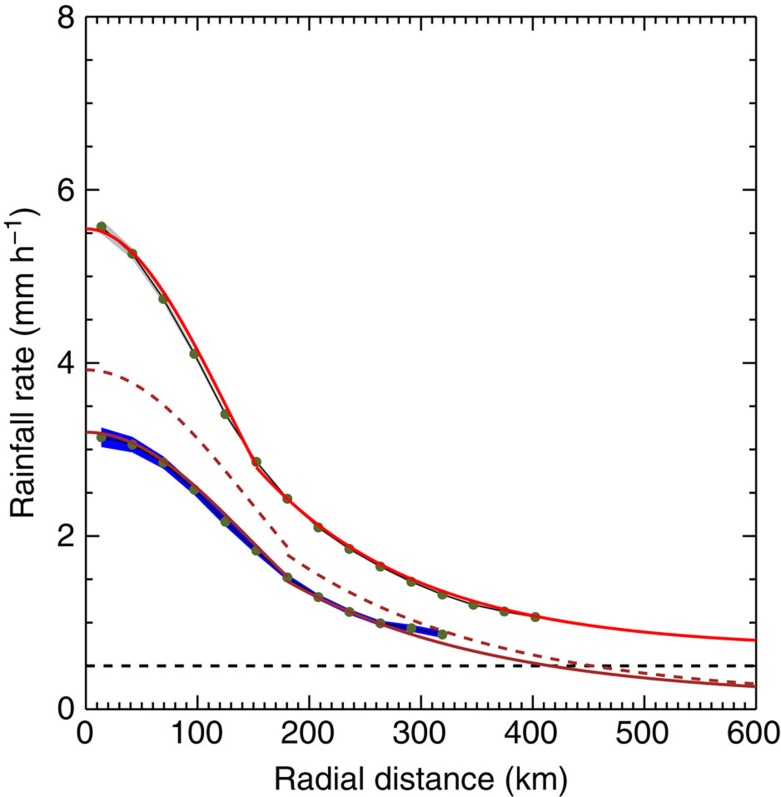
Expansion of TC rainfall radius with increasing relative SST. The solid brown and red lines indicate fits to the radial profile of rainfall rate of tropical storms in the [0 °C,1 °C] and [3 °C,4 °C] relative SST bins (original data shown in blue and grey). The brown dashed line shows the estimated increase in rainfall rate associated with a 3 °C increase in absolute SST from the [0 °C, 1 °C] distribution (assuming an increase of ~7.5% °n^–*n*^ and a fixed profile of vertical velocity). The black horizontal dotted line indicates the 0.5 mm h^−1^ rainfall rate threshold used to determine the rainfall radius.

The frequency distributions of TC rainfall radius based on all of these simulations are very similar (Fig. 4a). The overall increase in rainfall radius is only around 3 km for the P2K simulation (relative to the control simulation) and around 10 km for the P4K simulation (relative to the AMIP simulation). These small increases in rainfall radius are mainly due to increases in rainfall rate with increasing absolute SST (the rainfall rate threshold used in the method is fixed at 0.5 mm h^−1^ (see Fig. 5 and Methods). The negligible change in TC rainfall area under global warming scenarios is in sharp contrast to the strong dependence of TC size on absolute SST, and is consistent with the hypothesis that rainfall area depends primarily on relative SST. This suggests that, to the first order, TC rainfall area is primarily controlled by relative SST instead of absolute SST. Relative SST has also been identified as better than absolute SST for explaining variations in Atlantic TC frequency^5^,^25^,^26^ and tropical convection^27^.

In contrast to the negligible change in rainfall area, rainfall rate changes significantly among these experiments, both within 100 km of the TC centre (Fig. 4b) and within the TC rainfall area (Fig. 4c). Rainfall rate within 100 km of the TC centre increases by ~12% for the P2K experiment (relative to the control simulation) and ~25% for the P4K simulation (relative to the AMIP simulation). These increases decrease slightly to ~8% and ~18%, respectively, within the TC rainfall area. These increases in rainfall rate with absolute SST are consistent with previous studies^6^,^12^,^28^,^29^,^30^. Overall, the results suggest that TC rainfall rate is strongly impacted by absolute SST, while rainfall area is mainly controlled by relative SST.

## Discussion

The rainfall rate within a TC approximately equals the vertically integrated condensation of water vapour in the atmosphere, which is in turn proportional to the upward transport of water vapour out of the boundary layer^31^. Given a radial profile of vertical velocity *w(r)* at the top of the boundary layer, the rainfall rate is related to absolute *SST* as


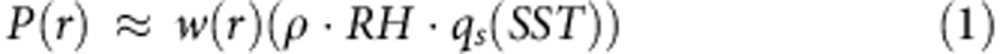


in which *P* is the rainfall rate, *r* is the distance from the centre of an axisymmetric hurricane, *q*_*s*_(*SST*) is saturation water vapour specific humidity at the given *SST*, *RH* is the average boundary layer RH and *ρ* is the air density. We find that we can reproduce the shape of the observed radial distribution of rainfall rate using a fit that exponentially decreases with the square of the radius near the centre and exponentially decreases with the radius beyond a certain radius (~ 150 km here) (Fig. 5). This approach is consistent with previous findings that the structure of a TC consists of an inner core and an outer region. The exponential decrease of vertical motion with the radius in the outer region is similar to previous work^14^,^15^,^16^,^32^. As absolute SST increases, if the TC wind field (and thus *w(r)*) does not change, the TC rainfall rate *P* will increase with SST following the Clausius–Clapeyron relationship. A 3 K increase in absolute SST would then increase the TC rainfall rate by ~22.5% and the TC size by ~20 km (assuming a fixed threshold of 0.5 mm h^−1^); the rainfall distribution would then follow the brown dashed line in Fig. 5. This change in TC size is much less than the ~100 km increase in TC size that results from a relative SST increase of 3 K (grey line in Fig. 5), and is consistent with the idea that TC size is largely insensitive to changes in absolute SST. The rainfall distribution will expand outwards as the relative SST increases (as shown by the red line in Fig. 5), so that the significant increase in rainfall area with increasing relative SST must be associated with an expansion of the TC wind field. This result is consistent with previous numerical simulations showing TC size increases as the environment becomes more humid^10^,^11^.

Previous modelling studies have suggested that TC size increases with increasing TC environmental RH due to the production of potential vorticity by active convection located at greater distance from the storm centre^10^,^11^,^33^. Free tropospheric RH in the tropics is large over regions of warmer relative SSTs due to the prevalent convective moistening in these regions^34^. Relative SST is a good indicator of mid-tropospheric RH at longer time scales in the tropics. As a result, a TC moving into an area of warmer relative SST tends to encounter higher mid-tropospheric RH, which in turn tends to increase condensational heating and reduce evaporative cooling. Both changes promote the expansion of TC tangential wind, size and rainfall area^10^,^11^. This result can explain why TCs tend to be the largest in the western North Pacific, where the largest values of relative SST and mid-tropospheric RH in the tropics are located. A uniform increase of SST does not necessarily raise the mid-tropospheric RH. Mid-tropospheric RH is expected to remain roughly constant with warming^35^, so that TC size is largely insensitive to absolute SST. These inferences are consistent with our results regarding the dependence of TC size on relative SST.

**Figure 6:**
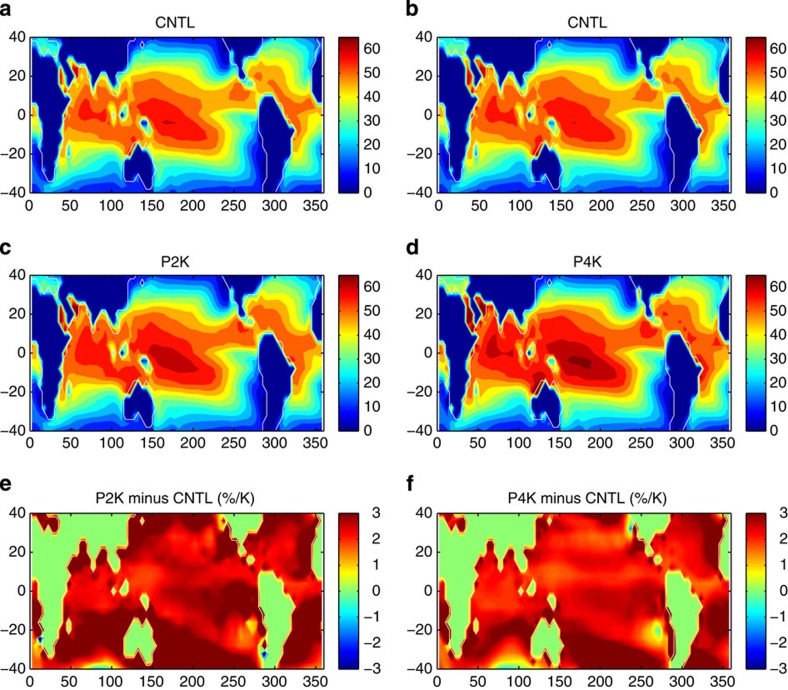
Potential intensity from HIRAM simulations. Geographical distribution of mean PI (m s^−1^) for the (**a**) control, (**b**) AMIP, (**c**) P2K and (**d**) P4K simulations, along with relative changes in PI (% K^−1^) between (**e**) the P2K and control simulations, and (**f**) the P4K and AMIP simulations.

Theoretical studies and idealized simulations suggest that PI can affect TC size^14^,^15^,^16^,^17^. PI is closely related to relative SST^25^.The strong dependence of TC rainfall area on relative SST might therefore indicate a dependence on PI. We attempt to distinguish the effects of relative SST from the effects of PI using the HIRAM simulations. In these simulations only the absolute SST is increasing while the relative SST is fixed, and thus they can be used to isolate the effects of absolute SST alone. As expected, PI increases in the warmer climate simulations (Fig. 6), with the global PI weighted by TC track frequency increasing by roughly 1.2 m s^−1^ K^−1^ in the P2K simulation (relative to the control simulation) and 1.0 m s^−1^ K^−1^ in the P4K simulation (relative to the AMIP simulation; Supplementary Fig. 3). This amounts to an increase of roughly 2.3–2.7% K^−1^ (global PI weighted by TC track frequency is 43 m s^−1^), four times larger than the simulated increase in rainfall area (~0.4–0.6% K^−1^) (Fig. 4). This result suggests that TC rainfall area may increase at a much slower rate than PI as the climate warms.

In contrast, if the increase in TC rainfall area in the warmer climate simulation is completely determined by changes in PI, then TC size should increase by 1.25–2.5 km for each 1 m s^−1^ increase in PI. The increase in PI with increasing relative SST reported by a previous study (~8 m s^−1^ K^–1^)^36^ suggests an increase in TC size of 10–20 km K^−1^, significantly less than the estimated using the results shown in Fig. 3 (30–40 km K^−1^). Relative SST therefore appears to be a better predictor of TC rainfall size than PI, although further investigations are needed to systematically quantify the roles of relative SST and PI on changes in TC size under global warming.

The above analyses lead us to conclude that changes in flooding induced by TCs under global warming will be caused primarily by increases in rainfall rate, rather than by increases in rainfall area. A significant increase in TC rainfall area is not expected unless the genesis locations and tracks of TCs shift to regions of higher relative SST or the spatial pattern of SST in the tropics changes.

## Methods

### Data

We use 11 years (1998–2008) of 3-hourly TRMM 3B42 precipitation data at 0.25° resolution^20^. TC track and intensities are taken from the 6-h International Best Track Archive for Climate Stewardship^22^ (IBTrACS) data, which combines data from multiple sources to provide a complete global climatology of TCs. A total of 1142 TCs occurred during the 11-year analysis period. We also use the global Cloud Archive User Services^21^ (CLAUS) satellite data set, which is based on ISCCP B3 data with additional quality control and satellite inter-calibration. Brightness temperatures at a resolution of one-third of a degree are used for the period 1985–2005. A total of 2,319 TCs occurred during this 21-year period. The absolute and relative SSTs at the TC positions are determined via linear interpolations from HadISST^37^ data at 1° resolution. The use of monthly SST data (rather than higher frequency SST data, such as daily SSTs) minimizes the effects of transient cooling of SSTs by TC induced mixing of the ocean surface layer. Environmental RH, which is relevant for TC rainfall area, also varies relatively slowly over the tropics.

### Model simulations

Four HIRAM simulations are used. HIRAM produces realistic simulations of TC statistics, including the global climatology and variations at seasonal and interannual time scales. The first pair of HIRAM simulations consists of a 25-year (1981–2005) control simulation using climatological SSTs and sea ice (seasonal variations are included but interannual variations are not) and a 25-year companion simulation in which a uniform 2 K warming is imposed (P2K). The second pair of HIRAM simulations consists of a 29-year (1981–2009) Atmospheric Model Intercomparison Project (AMIP) type simulation using historical monthly SSTs and sea ice and a companion simulation in which a uniform 4 K warming is imposed (P4K).

### TC rainfall size determination

The calculation of TC rainfall area requires objective methods to determine the region that contains most of the TC rainfall. TCs generally possess an annular structure in their inner cores, although rain bands can sometimes distort this axisymmetry. The first method, which is applied to the TRMM observations and the model simulations, assumes that TC rainfall is axisymmetric and a varying radius covering most of the TC rainfall is determined. To increase the sample size and fully utilize the 3-hourly TRMM data, we determine the 3-h position of each TC by linearly interpolating the 6-h TC positions from the IBTrACS. At each TC instance, TRMM data within a 30° × 30° region centred at the TC center (121 × 121 grid cells) are prepared first. The 2-dimensional data are then converted to a polar projection and azimuthally averaged rainfall is computed in 0.25° bins. The rainfall radius is determined by the following three criteria: average rainfall greater than a threshold value (here, 0.5 mm h^−1^); fractional s.d. <1.0 (large variance indicates strong asymmetry in the precipitation field, possibly due to mesoscale convective systems near the TC); and a radial gradient of average rainfall <0.2 (average rainfall generally decreases quickly away from the storm centre, so that this criterion can be used to separate TC rainfall from nearby rainfall not directly associated with the TC). Supplementary Figure 4a shows an example of this methodology for TC Heta in the South Pacific. The method is able to capture the overall rainfall area, specifically the TC inner core and the primary and secondary rainbands. The exact value of the radius is sensitive to the parameters used in the criteria, but this sensitivity does not affect our results so long as all TCs are analysed using the same objective method. This method is also used to determine the rainfall areas and radii of TCs simulated by the HIRAM model. As shown in Fig. 5, rainfall radius can increase simply because of an increase of rainfall rate with increasing absolute SST without a change in the TC wind field. To evaluate the impacts of changes in absolute SST on the rainfall radius, we reduce TC rainfall rates in the P2K simulation by 15% and in the P4K simulation by 30% (approximately following the Clausius–Clapeyron scaling) before applying the method. The mean rainfall size in the P2K and P4K simulations after this scaling is slightly smaller (by ~3 km) than the mean rainfall size in the control and AMIP simulations.

Another objective method is used to identify TC rainfall area based on CLAUS brightness temperatures. This method accounts for persistence and asymmetries in the distribution of TC rainfall. The method is based on a region of interest (ROI) image processing algorithm, which automatically determines the closed blob of rainfall associated with a TC. First, brightness temperature data within a 30° × 30° area centred on the TC position is prepared. Second, all ROIs within this area are identified and labelled, using a threshold brightness temperature of 219 K (which corresponds well to deep raining convective clouds)^38^. ROIs with populations >15 pixels (~19,000 km^2^) located within a radius of 2° from the TC centre are assigned to be the TC rainfall field. Only one ROI is identified in ~98% of the cases and this ROI is then used to estimate TC rainfall area. In the other ~2% of the cases, either zero or two (or more) ROIs are identified and a missing value is recorded for these cases. The area equivalent radius is computed by summing the pixel areas (converted from latitude-longitude to distance) within the ROI. The rainfall area of TC Heta determined using this method covers the inner core and the primary rainband (Supplementary Fig. 4b). This method generally gives a smaller radius than the first method. The brightness temperature field is spatially more continuous, and therefore more suitable for the ROI method. The TRMM rainfall field is spatially heterogeneous, so that the ROI method easily fails.

We composite the radius, rainfall radial distribution and 2-D rainfall field based on the intensity and the absolute and relative SSTs associated with each TC (where the latter two are linearly interpolated in both time and space from monthly mean HadISST data to the TC centre). Using average SSTs within 100 km of the TC centre gives similar results. Only storms over ocean pixels within the tropics (30°S–30°N) are included in the composite to exclude complications with landfall or interactions with mid-latitude synoptic systems.

## Author contributions

All authors contributed to the central ideas presented in the paper. Y. L. performed the analysis and wrote the paper. M. Z. conducted model simulations. All authors discussed the results and edited the manuscript.

## Additional information

**How to cite this article:** Lin, Y. *et al*. Tropical cyclone rainfall area controlled by relative sea surface temperature. *Nat. Commun.* 6:6591 doi: 10.1038/ncomms7591 (2015).

## Supplementary Material

Supplementary Information Supplementary Figures 1-4

## References

[b1] Rappaport E. N. Loss of life in the United States associated with recent Atlantic tropical cyclones. Bull. Am.. Meteor. Soc. 81, 9 2065–2074 (2000) .

[b2] Jiang H. & Zipser E. J. Contribution of tropical cyclones to the global precipitation from eight seasons of TRMM data: Regional, seasonal, and interannual variations. J. Climate 23, 1526–1543 (2010) .

[b3] Sugi M., Murakami H. & Yoshimura J. A reduction in global tropical cyclone frequency due to global warming. SOLA 5, 164–167 (2009) .

[b4] Bender M. A. . Modeled impact of anthropogenic warming of the frequency of intense Atlantic hurricanes. Science 327, 454–458 (2010) . 20093471 10.1126/science.1180568

[b5] Zhao M. & Held I. M. TC-Permitting GCM simulations of hurricane frequency response to sea surface temperature anomalies projected for the late-twenty-first century. J. Climate 25, 2995–3009 (2012) .

[b6] Knutson T. R. . Tropical cyclones and climate change. Nat. Geosci. 3, 157–163 (2010) .

[b7] Weatherford C. L. & Gray W. M. Typhoon structure as revealed by aircraft reconnaissance. Part I: Data analysis and climatology. Mon. Wea. Rev. 116, 1032–1043 (1988) .

[b8] Matyas C. J. Association between the size of hurricane rain fields at landfall and their surrounding environments. Meteorol. Atmos. Phys. 106, 135–148 (2010) .

[b9] Merrill R. T. A comparison of large and small tropical cyclones. Mon. Wea. Rev. 112, 1408–1418 (1984) .

[b10] Wang Y. How do outer spiral rainbands affect tropical cyclone structure and intensity? J. Atmos. Sci. 66, 1250–1273 (2009) .

[b11] Hill K. A. & Lackmann G. M. Influence of environmental humidity on tropical cyclone size. Mon. Wea. Rev. 137, 3294–3315 (2009) .

[b12] Hill K. A. & Lackmann G. M. The impact of future climate change on TC intensity and structure: A downscaling approach. J. Climate 24, 4644–4661 (2011) .

[b13] Quiring S., Schumacher A. C., Labosier C. & Zhu L. Variations in mean annual tropical cyclone size in the Atlantic. J. Geophys. Res. 116, D09114 (2011) .

[b14] Emanuel K. A. An air-sea interaction theory for tropical cyclones. Part I: Steady state maintenance. J. Atmos. Sci. 43, 585–604 (1986) .

[b15] Emanuel K. A. & Rotunno R. Self-stratification of tropical cyclone outflow. Part I: Implications for storm structure. J. Atmos. Sci. 68, 2236–2249 (2011) .

[b16] Chavas D. R. & Emanuel K. A. Equilibrium tropical cyclone size in an idealized state of axisymmetric radiative-convective equilibrium. J. Atmos. Sci. 71, 1663–1680 (2014) .

[b17] Khairoutdinov M. & Emanuel K. A. Rotating radiative-convective equilibrium simulated by a cloud-resolving model. J. Adv. Model. Earth Sys. 5, 816–825 (2013) .

[b18] Chavas D. R. & Emanuel K. A. A QuickSCAT climatology of tropical cyclone size. Geophys. Res. Lett. 37, L18816 (2010) .

[b19] Knaff J. A., Longmore S. P. & Molenar D. A. An objective satellite-based tropical cyclone size climatology. J. Climate 27, 455–476 (2014) .

[b20] Huffman G. J. . The TRMM multi-satellite precipitation analysis: quasi-global, multi-year, combined-sensor precipitation estimates at fine scale. J. Hydrometeor. 8, 38–55 (2007) .

[b21] Hodges K., Chappell D. W., Robinson G. J. & Yang G. An improved algorithm for generating global window brightness temperatures from multiple satellite infra-red imagery. J. Atmos.Ocean Technol. 17, 1296–1312 (2000) .

[b22] Knapp K. R., Kruk M. C., Levinson D. H., Diamond H. J. & Neumann C. J. The International Best Track Archive for Climate Stewardship (IBTrACS): unifying tropical cyclone best track data. Bull. Am. Meteor. Soc. 91, 363–376 (2010) .

[b23] Zhao M., Held I. M., Lin S.-J. & Vecchi G. A. Simulations of global hurricane climatology, interannual variability, and response to global warming using a 50 km resolution GCM. J. Climate 22, 6653–6678 (2009) .

[b24] Held I. M. & Zhao M. The response of tropical cyclone statistics to an increase in co2 with fixed sea surface temperatures. J. Clim. 24, 5353–5364 (2011) .

[b25] Vecchi G. A. & Soden B. J. Effect of remote sea surface temperature change on tropical cyclone potential intensity. Nature 450, 1066–1070 (2007) . 18075590 10.1038/nature06423

[b26] Vecchi G. A., Swanson K. L. & Soden B. J. Whither hurricane activity. Science 322, 687–689 (2008) . 18974337 10.1126/science.1164396

[b27] Johnson N. C. & Xie S. P. Changes in the sea surface temperature threshold for tropical convection. Nat. Geosci. 3, 842–845 (2010) .

[b28] Emanuel K. A. & Sobel A. Response of tropical sea surface temperature, precipitation, and tropical cyclone‐related variables to changes in global and local forcing. J. Adv. Model. Earth Sys. 5.2, 447–458 (2013) .

[b29] Knutson T. R. . Dynamical downscaling projections of twenty-first-century atlantic hurricane activity: CMIP3 and CMIP5 model-based scenarios. J. Clim. 26, 6591–6617 (2013) .

[b30] Villarini G. . Sensitivity of tropical cyclone rainfall to idealized global scale forcings. J. Climate 27, 4622–4641 (2014) .

[b31] Langousis A. & Veniele V. Theoretical model of rainfall in tropical cyclones for the assessment of long-term risk. J. Geophys. Res. 114, D02106 (2008) .

[b32] Emanuel K. A tropical cyclone energetics and structure. in Atmos. Turbulence Mesoscale Meteorol eds Fedorovich E., Rotunno R., Stevens B. Cambridge University Press280 (2004) .

[b33] Sun Y., Zhong Z., Yi L., Ha Y. & Sun Y. The opposite effects of inner and outer sea surface temperature on tropical cyclone intensity. J. Geophys. Res. 2193–2208 (2014) .

[b34] Bretherton C. S., Peters M. E. & Back L. E. Relationships between water vapor path and precipitation over the tropical oceans. J. Climate 17, 1517–1528 (2004) .

[b35] Rappin E. D., Nolan D. S. & Emanuel K. A. Thermodynamic control of tropical cyclogenesis in environments of radiative‐convective equilibrium with shear. Quart. J. R. Meteor. Soc. 136, 1954–1971 (2010) .

[b36] Ramsay H. A. & Sobel A. H. Effects of relative and absolute sea surface temperature on tropical cyclone potential intensity using a single-column model. J. Climate 24, 183–193 (2011) .

[b37] Rayner N. A. . Global analyses of sea surface temperature, sea ice, and night marine air temperature since the late nineteenth century. J. Geophys. Res. 108, 4407 (2003) .

[b38] Evans J. L. & Shemo R. E. A procedure for automated satellite-based identification and climatology development of various classes of organized convection. J. Appl. Meteor. 35, 638–652 (1996) .

