# Supplementary material for: Tropical cyclone rainfall area controlled by relative sea surface temperature
Source: Nat Commun. 2015 Mar 12;6:6591. doi: 10.1038/ncomms7591 (PMC4382685; doi:10.1038/ncomms7591)
Supplement: Supplementary Information — Supplementary Figures 1-4 [file ncomms7591-s1.pdf]

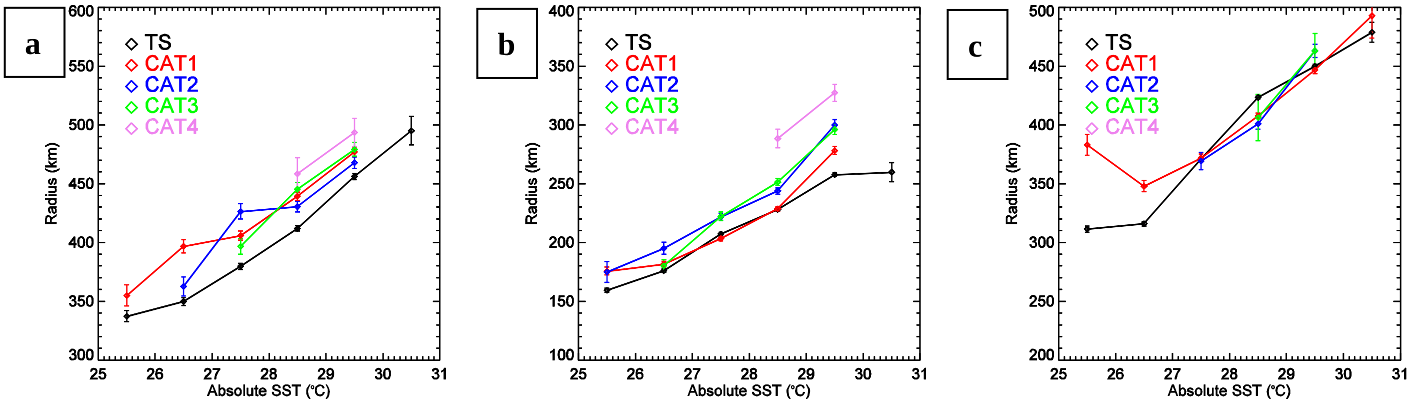

Supplementary Figure 1. **Variation of tropical cyclone rainfall radius with absolute SST.** (a) Average tropical cyclone rainfall radii in the specified 1-degree absolute SST bins based on TRMM data using the first method (see Methods for details). Different colors indicate sets of tropical cyclones of different intensities, from tropical storm (TS) to category 4 (CAT4). (b) Same as **a**, but based on CLAUS data using the second method. (c) Same as **a**, but based on the HIRAM AMIP simulation using the first method. Error bars indicate one standard error of the mean.

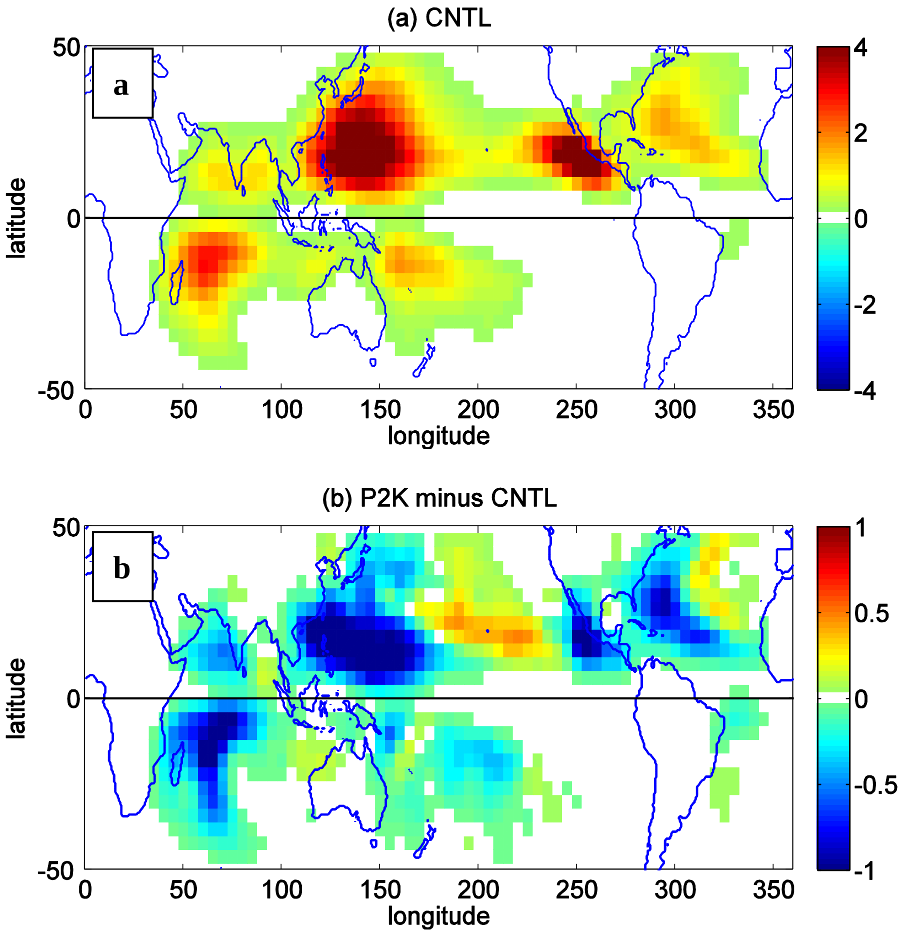

Supplementary Figure 2. **TC track density from HIRAM simulations.** (a) Geographical distribution of the TC track density (unit: annual count per 4°x5^{o} (latitude-longitude) area) of the control simulation. (b) Geographical distribution of TC track density differences (the P2K minus the control simulation).

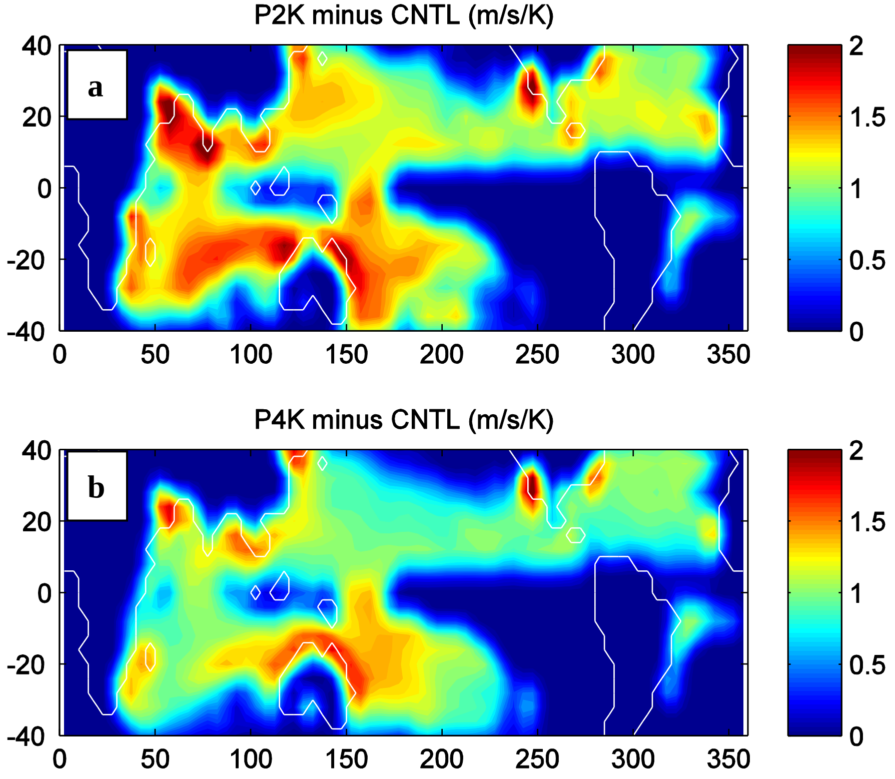

Supplementary Figure 3. Changes of potential intensity (PI) weighted by tropical cyclone track frequency. (a) Geographical distribution of relative changes in PI (m s^{-1} K^{-1}, weighted by TC track frequency) between the P2K and control simulations. (b) Same as **a**, but between the P4K and AMIP simulations.

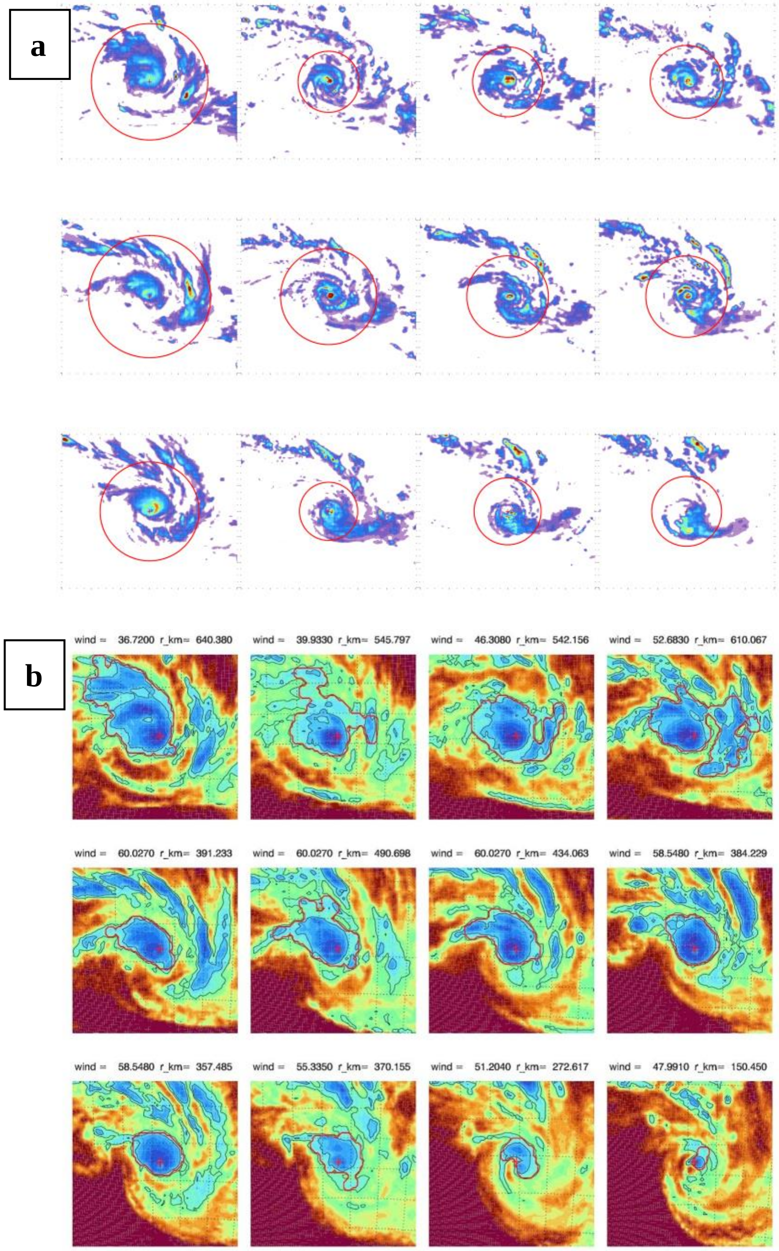

Supplementary Figure 4. **An example for the two methods.** (a) Snapshots of rainfall field from the TRMM data associated with tropical cyclone Heta in the South Pacific. Red circles denote the rainfall area determined using the first method. (b) Same as **a**, except for snapshots of cloud brightness temperatures from the CLAUS data. Red contours denote the rainfall area determined using the second method.
